# Opioid utilization after orthopaedic trauma hospitalization among Medicaid-insured adults

**DOI:** 10.3389/fpubh.2024.1327934

**Published:** 2024-03-26

**Authors:** Nicholas A. Giordano, Guantao Zhao, Manvitha Kalicheti, Mara L. Schenker, Yolanda Wimberly, Cammie Wolf Rice, Nicoleta Serban

**Affiliations:** ^1^Nell Hodgson Woodruff School of Nursing, Emory University, Atlanta, GA, United States; ^2^H. Milton Stewart School of Industrial and Systems Engineering, Georgia Institute of Technology, Atlanta, GA, United States; ^3^Department of Orthopaedics, School of Medicine, Emory University, Atlanta, GA, United States; ^4^Grady Memorial Hospital, Atlanta, GA, United States; ^5^The Christopher Wolf Crusade, Atlanta, GA, United States

**Keywords:** opioid, trauma, injury, pain, emergency medicine, orthopaedic trauma & surgery

## Abstract

Opioids are vital to pain management and sedation after trauma-related hospitalization. However, there are many confounding clinical, social, and environmental factors that exacerbate pain, post-injury care needs, and receipt of opioid prescriptions following orthopaedic trauma. This retrospective study sought to characterize differences in opioid prescribing and dosing in a national Medicaid eligible sample from 2010–2018. The study population included adults, discharged after orthopaedic trauma hospitalization, and receiving an opioid prescription within 30 days of discharge. Patients were identified using the International Classification of Diseases (ICD-9; ICD-10) codes for inpatient diagnosis and procedure. Filled opioid prescriptions were identified from National Drug Codes and converted to morphine milligram equivalents (MME). Opioid receipt and dosage (e.g., morphine milligram equivalents [MME]) were examined as the main outcomes using regressions and analyzed by year, sex, race/ethnicity, residence rurality-urbanicity, and geographic region. The study population consisted of 86,091 injured Medicaid-enrolled adults; 35.3% received an opioid prescription within 30 days of discharge. Male patients (OR = 1.12, 95% CI: 1.07–1.18) and those between 31–50 years of age (OR = 1.15, 95% CI: 1.08–1.22) were found to have increased odds ratio of receiving an opioid within 30 days of discharge, compared to female and younger patients, respectively. Patients with disabilities (OR = 0.75, 95% CI: 0.71–0.80), prolonged hospitalizations, and both Black (OR = 0.87, 95% CI: 0.83–0.92) and Hispanic patients (OR = 0.72, 95% CI: 0.66–0.77), relative to white patients, had lower odds ratio of receiving an opioid prescription following trauma. Additionally, Black and Hispanic patients received lower prescription doses compared to white patients. Individuals hospitalized in the Southeastern United States and those between the ages of 51–65 age group were found to be prescribed lower average daily MME. There were significant variations in opioid prescribing practices by race, sex, and region. National guidelines for use of opioids and other pain management interventions in adults after trauma hospitalization may help limit practice variation and reduce implicit bias and potential harms in outpatient opioid usage.

## 1 Introduction

Pain after orthopaedic injury is complex, pervasive, and debilitating when undermanaged. Orthopaedic trauma is a leading form of injury in America, with 21% of injured patients requiring hospitalization (1). Up to one-third of patients report experiencing chronic pain months after discharge following orthopaedic injury (2, 3). Optimizing acute pain after orthopaedic injury is vital to attenuating the pain response, mitigating the development of chronic pain, and reducing psychological symptom severity (2, 4, 5). Given pain is the common complaint for individuals seeking emergency department (ED) care (6, 7), adequately addressing patients’ pain management needs after orthopaedic injury may impact subsequent care outcomes and opioid utilization.

Opioids are the mainstay of pain management. Guidelines recommend clinicians prescribe opioid medications to optimize acute pain following orthopaedic trauma (8–10). While declines in opioid prescribing to manage pain have been observed in other care settings and specialties, orthopaedic trauma care pain management continues to be centered around opioids (11, 12). Up to two-thirds of patients require an opioid refill after undergoing surgery following orthopaedic trauma (13). Yet access to opioid medications throughout recovery, when pain can still persist, is not equitable. Substantial differences in opioid prescribing and dosing have been noted across other patient populations based on race and geographic locations (14–16). Despite the prevalence of orthopaedic trauma and subsequent burden of pain, there remains a dearth of research elucidating national prescribing trends and potential differences.

Nationally representative data on opioid prescribing and utilization after orthopaedic trauma are needed to discern demographic and clinical factors that may influence prescribing. Previously, investigations have sought to elucidate opioid prescribing trends and care utilization in older adults and privately-insured general orthopaedic patients (14, 17). The preponderance of studies examining opioid prescribing and pain needs after orthopaedic injury have been conducted at single care centers, with few larger database studies focusing exclusively on single site fractures or joint replacements (12, 18–20). Less is known regarding prescribing after orthopaedic trauma. This study sought to characterize differences in opioid prescribing and dosing over a 30 and 90 day period in a national Medicaid eligible sample following hospitalization due to orthopaedic trauma. It was hypothesized that differences in prescribing patterns would be noted based on patient clinical characteristics and demographics.

## 2 Methods

### 2.1 Data source

The primary data source consists of 2010–2018 Medicaid claims data acquired from the Centers for Medicare and Medicaid Services, including identifiable individual-level claims with information on service utilization for all Medicaid-enrolled beneficiaries. Included for each claim were data entries specifying the identification of each Medicaid enrollee, demographics, service date, the International Classification of Diseases (ICD-9, ICD-10) codes, Current Procedural Terminology codes, National Drug Codes (NDCs) along with days of supply, and dosage. Using CDC Opioid MME Conversion Factors (12, 20, 21) we translated NDCs to obtain information about their corresponding drugs.

All data derived from the Medicaid files meet a minimum cell size of 11 enrollees according to the Data Use Agreement. This study was approved by the Institutional Review Board of Georgia Institute of Technology (protocol #H11287).

### 2.2 Study population

The study population included national Medicaid enrollees ages 18–65 with a trauma-related inpatient visit. We used the inpatient claims to identify Medicaid enrollees with trauma-related diagnosis codes (Supplementary Tables S1–S3). Since some patients had multiple trauma-related records, we included those with up to two trauma-related hospitalizations to exclude patients with severe repeated trauma. We excluded pregnant women and Medicaid enrollees not enrolled 30 days after discharge from the study population. Rural-Urban Continuum Codes (RUCC) specified the rurality-urbanicity of the patients’ residence. Demographics including age, race/ethnicity, region, Medicaid eligibility and sex were extracted.

### 2.3 Opioid prescribing outcomes

Opioid outcomes were based on published CDC guidelines and previous research to evaluate opioid use (4, 22, 23). All opioid claims of each patient in the study population were considered throughout a 30 day or 90 day period after the trauma-related inpatient discharge date, called herein *opioid-monitoring period*. The individual-level outcomes derived over the opioid-monitoring period included:

1. *Filled-prescription outcome*: a binary indicator specifying whether an opioid prescription was recorded.
2. *Opioid dosage outcome*: sum of Morphine milligram equivalents (MME) across all prescriptions divided by the number of prescription days (MED).

### 2.4 Explanatory factors

Explanatory factors included length of hospitalization in days, as a proxy of trauma severity, history of traumatic hospitalization, year of injury, age, sex, race/ethnicity, rurality-urbanicity of beneficiary residence, region where treatment was delivered (e.g., Southeastern, etc.) and Medicaid eligibility criteria (e.g., disability, income-based).

### 2.5 Statistical analysis

Logistic regression was used to examine how the explanatory factors were associated with the odds ratio of opioid prescription filled within the opioid-monitoring period. Linear regression models examined factors linked to variability in the log of the MED for patients with recorded opioid use within the opioid-monitoring period. To focus on patients with appropriate dosages, we excluded individuals with a MED greater than or equal to 90 defined as those as being in the “very high risk” category as outlined by CDC guidelines (24). To improve the distributional properties of the linear regression, a log transformation was applied to the MED values.

To correct for “inflated” statistical significance due to large sample size (25, 26), we applied the regression models to 100 different sub-samples, each sub-sample consisting of 30% subsample of the study population. The number of significant *p*-values (*p* ≤ 0.05) was counted for each predictor across the 100 replicates. Statistical significance was established if 90% or more of the *p*-values in the 100 model replicates showed significance. We present the mean odds ratio (opioid use) and predicted mean (opioid dosage) across these 100 model replicates. The 95% confidence interval (CI) for the regression coefficients was derived using the 2.5th percentile for the lower bound and the 97.5th percentile for the upper bound of each transformed regression coefficient (e.g., odds ratio) across the model replicates.

## 3 Results

A total of 86,091 Medicaid-enrolled adults were identified as having had an eligible trauma-related hospitalization during the study period (Table 1). Among them, 30,361 (35.3%) patients received an opioid prescription <30 days after discharge, and 36,553 (42.5%) patients received an opioid prescription <90 days after discharge (Supplementary Table S4). Notably, the demographic distribution revealed that patients aged 51–65 constituted a significant proportion, accounting for 39,167 individuals (45.5%), while female patients represented 44,077 cases (51.2%) receiving an opioid. Furthermore, most patients identified as white (49.9%). The average MED was 8.4 (SD: 9.2) and the average total MME prescribed was 109.3 (SD: 160.0).

**Table 1:** Sample characteristics.

	Total *N* = 86,091	Opioid prescription *N* = 37,020	No opioid prescription *N* = 49,071	*p*-value^a^
Age, y, *n* (%)				
18–30 **(Base)**	14,241 (16.54)	6,157 (16.63)	8,084 (16.47)	0.544
31–50	32,683 (37.96)	15,177 (41.00)	17,506 (35.67)	<0.001
51–65	39,167 (45.50)	15,686 (42.37)	23,481 (47.85)	<0.001
Sex, *n* (%)				
Female **(Base)**	44,077 (51.20)	18,308 (49.45)	25,769 (52.51)	<0.001
Male	42,014 (48.80)	18,712 (50.55)	23,302 (47.49)	<0.001
Race/ethnicity, *n* (%)				
White **(Base)**	42,946 (49.88)	19,021 (51.38)	23,925 (48.76)	<0.001
Black	17,076 (19.83)	7,085 (19.14)	9,991 (20.36)	<0.001
Hispanic	9,655 (11.21)	3,592 (9.70)	6,063 (12.36)	<0.001
Other	3,547 (4.12)	1,460 (3.94)	2087 (4.25)	0.025
Rurality-Urbanicity, *n* (%)				
Metro counties **(Base)**	66,402 (77.13)	28,022 (75.69)	38,380 (78.21)	<0.001
Nonmetro counties	14,466 (16.80)	6,395 (17.27)	8,071 (16.45)	0.001
Region, *n* (%)				
Midwest **(Base)**	19,522 (22.68)	7,538 (20.36)	11,984 (24.42)	<0.001
Southeast	16,706 (19.41)	7,063 (19.08)	9,643 (19.65)	0.036
Northeast	21,646 (25.14)	10,320 (27.88)	11,326 (23.08)	<0.001
West	18,844 (21.89)	8,098 (21.87)	10,746 (21.90)	0.939
Southwest	9,373 (10.88)	4,001 (10.81)	5,372 (10.95)	0.522
Year of hospitalization, *n* (%)				
2010–2012 **(Base)**	23,368 (27.14)	7,014 (18.95)	16,354 (33.33)	<0.001
2013–2014	10,188 (11.83)	4,233 (11.43)	5,955 (12.14)	0.002
2015–2016	21,840 (25.37)	10,740 (29.01)	11,100 (22.62)	<0.001
2017–2018	30,695 (35.66)	15,033 (40.61)	15,662 (31.92)	<0.001
Length of stay, d, mean (SD)	7.03 (7.17)	6.77 (6.51)	7.23 (7.63)	0.999
Eligibility criteria, *n* (%)				
Income-based **(Base)**	52,507 (60.99)	25,459 (68.77)	27,048 (55.12)	<0.001
Disability	33,584 (39.01)	11,561 (31.23)	22,023 (44.88)	<0.001
History of traumatic hospitalization, *n* (%)	4,563 (5.30)	2040 (5.51)	2,523 (5.14)	0.017

SD, standard deviation.

a In the context of a two-sample proportion test (two separate samples were compared to ascertain if their proportions showed a significant difference). A significance level of 95 indicated that the conclusions were intended to maintain a 95 confidence level.

### 3.1 Opioid prescription receipt

Differences in prescribing were observed based on patient race (Table 2). Compared to the white patients, Black patients had lower odds of having a prescription filled <30 days after discharge (OR = 0.87, 95% CI: 0.83–0.92). This indicates that Black patients had 13% lower odds of the outcome compared to white patients. Similarly, Hispanic patients had 28% lower odds of filling a prescription compared to white patients (OR = 0.72, 95% CI: 0.66–0.77). Individuals categorized as “other” race did not statistically differ in receipt of opioid compared to white patients (OR = 0.86, 95% CI: 0.77–0.96).

**Table 2:** Estimated odds ratios and their statistical inference using the multivariable logistic regression for the opioid use binary outcome analysis with after discharge one month opioid-monitoring period (*N* = 86,091).

	Odds ratio	95 CI lower bound	95 CI upper bound	Percent of *p*-values <0.05^a^
Age, years				
18–30 **(Base)**	–	–	–	–
31–50	1.15	1.08	1.22	**97**
51–65	0.99	0.92	1.05	4
Sex				
Female **(Base)**	–	–	–	–
Male	1.12	1.07	1.18	**100**
Race/ethnicity				
White **(Base)**	–	–	–	–
Black	0.87	0.83	0.92	**99**
Hispanic	0.72	0.66	0.77	**100**
Other	0.86	0.77	0.96	57
Rurality-Urbanicity				
Metro counties **(Base)**	–	–	–	–
Nonmetro counties	1.10	1.04	1.18	81
Region				
Midwest **(Base)**	–	–	–	–
Southeast	0.95	0.88	1.01	25
Northeast	0.97	0.91	1.04	12
West	1.01	0.94	1.08	3
Southwest	1.00	0.92	1.09	2
Year of hospitalization				
2010–2012 **(Base)**	–	–	–	–
2013–2014	1.56	1.44	1.71	**100**
2015–2016	1.94	1.81	2.10	**100**
2017–2018	2.03	1.88	2.22	**100**
Length of stay, days	0.99	0.99	0.99	**97**
Eligibility criteria				
Income-based **(Base)**				
Disability	0.75	0.72	0.79	**100**
History of traumatic hospitalization	1.12	0.99	1.24	34

CI, confidence interval. Bold *p*-value indicates statistical significance.

a A logistic regression was used to examine how the explanatory factors explain the odds ratio of opioid prescription filled versus not filled within the opioid-monitoring period. To correct for “inflated statistical significance” due to a large sample size, the regression was estimated in 100 sub-samples that included 30% of the population. The mean odds ratio is presented from these 100 replicates, and the 95% confidence interval is derived using the 2.5th percentile for the lower bound and the 97.5th percentile for the upper bound of the estimated odds ratio across the 100 model replicates. The percent of *p*-values for each covariate that were significant in these 100 sub-samples is presented in the far-right column.

Patient demographics, including age, gender, and Medicaid eligibility were found to be linked to receipt of an opioid prescription after injury. Compared to those between ages 18–30, individuals aged 31–50 had higher odds of filling a prescription <30 days after discharge (OR = 1.15, 95% CI: 1.08–1.22). Alternatively, being between the ages of 51–65 was not significantly associated with having a prescription filled <30 days after discharge (OR = 0.99, 95% CI: 0.92–1.05). Male Medicaid beneficiaries had increased odds of having a prescription filled <30 days after discharge, 12%, compared to females (OR = 1.12, 95% CI: 1.07–1.18). Individuals eligible for Medicaid due to disability were observed with lower odds ratio for opioid receipt < 30 days (OR = 0.75, 95% CI: 0.71–0.80). This indicates that individuals who qualified for eligibility based on disability had lower odds of having a prescription filled <30 days after discharge compared to those who were Medicaid eligible based on income.

Further, year of injury was associated with odds of opioid receipt. Patients injured between 2013–2014 had higher odds of having a prescription filled <30 days after discharge (OR = 1.56, 95% CI: 1.44–1.71). This indicates that compared to 2010–2012, patients hospitalized from 2013–2014 were associated with an increased odds of receiving an opioid. Similarly, the years 2015–2016 (OR = 1.94, 95% CI: 1.81–2.10) and 2017–2018 (OR = 2.03, 95% CI: 1.88–2.22) were also significantly associated with higher odds of having a prescription filled <30 days after discharge.

No significant difference in the odds of having a prescription filled <30 days after discharge were observed between the urbanicity categories (OR = 1.10, 95% CI: 1.04–1.18). Similarly, no differences in opioid receipt were noted by region compared to the Midwest. Previous traumatic injury requiring hospitalization was not associated with receipt of prescription (OR = 1.11, 95% CI: 0.99–1.24).

Similar trends were noted in the 90 day models for opioid prescription receipt (Supplementary Tables S5–S7).

### 3.2 Opioid dosage analysis: 1 month period

MED differed by race in the study population (Table 3). Black patients received lower doses, on average −0.10 log-transformed MED, than white patients (95% CI: −0.16, −0.05). Similarly, Hispanic patients were found to receive significantly lower MED compared to white patients (*β* = −0.18, 95% CI: −0.24, −0.11). No differences were observed in MED between white patients and patients who identified as “other” races.

**Table 3:** Estimated coefficients and their statistical inference using log-transformed linear regression of the opioid dosage outcome with recorded opioid use within the one-month opioid-monitoring period (*N* = 30,361).

	Coefficients	95% CI lower bound	95% CI upper bound	Percent of *p*-values <0.05^a^
Age, years				
18–30 **(Base)**	–	–	–	–
31–50	−0.07	−0.11	−0.02	55
51–65	−0.19	−0.24	−0.14	**100**
Sex				
Female **(Base)**	–	–	–	–
Male	0.05	0.00	0.09	41
Race/ethnicity				
White **(Base)**	–	–	–	–
Black	−0.10	−0.16	−0.05	**93**
Hispanic	−0.18	−0.24	−0.11	**100**
Other	0.07	−0.02	0.18	18
Rurality-Urbanicity				
Metro counties **(Base)**	–	–	–	–
Nonmetro counties	−0.02	−0.06	0.03	5
Region				
Midwest **(Base)**	–	–	–	–
Southeast	−0.11	−0.17	−0.06	**93**
Northeast	0.14	0.09	0.19	**100**
West	0.03	−0.04	0.09	8
Southwest	−0.06	−0.12	0.01	19
Year of hospitalization				
2010–2012 **(Base)**	–	–	–	–
2013–2014	0.06	−0.03	0.15	26
2015–2016	0.14	0.07	0.22	**99**
2017–2018	0.25	0.18	0.32	**100**
Length of stay	−0.01	−0.01	0.01	18
Eligibility criteria				
Income-based **(Base)**	–	–	–	–
Disability	−0.05	−0.11	−0.01	33
History of traumatic hospitalization	0.01	−0.07	0.09	2

CI, confidence interval. Bold *p*-value indicates statistical significance. The coefficients is presented from these 100 replicates, and the 95% confidence interval is derived using the 2.5th percentile for the lower bound and the 97.5th percentile for the upper bound of the predicted difference in visits across the 100 model replicates. The percent of *p*-values for each covariate that were significant in these 100 sub-samples is presented in the far-right column.

a Linear regression was used to examine how the explanatory factors explain the variability in the log of the average MME per-day for patients with recorded opioid use within the opioid-monitoring period. To correct for inflated statistical significance’ due to a large sample size, the regression was estimated in 100 sub-samples that included 30% of the population.

Differences in opioid dosing were noted across patient age groups, patients’ residence regions, and the year of injury. Compared to patients 18–30 years of age, individuals between 51–65, received a prescription dose of −0.19 log-transformed MED (95% CI: −0.24, −0.14). No differences were observed between those 31–50 and those between 18–30 years of age. Patients in the Southeast received lower prescription doses compared to the Midwest (*β* = −0.11, 95% CI: −0.17, −0.06). Conversely, patients in the Northeast were found to have higher log-transformed MED compared to the Midwest (*β* = 0.14, 95% CI: 0.09, 0.19). No differences in MED were observed between the Western and the Southwestern regions compared to the Midwest. From 2015 to 2018, patients received higher MED compared to the reference group of 2010–2012, with patients seen in 2015–2016 receiving an average of 0.14 higher MED (95% CI: 0.07, 0.22) and those seen between 2017–2018 receiving even higher doses (*β* = 0.25, 95% CI: 0.18, 0.32). No differences in MED were seen by gender, urbanicity, length of stay, Medicaid eligibility, nor based on history of traumatic hospitalization.

Similar trends were observed in the 90 days model (Supplementary Table S8).

## 4 Discussion

In this national sample of Medicaid eligible patients hospitalized following orthopaedic trauma, over a third of patients received an opioid prescription within 30 days after discharge, and 42.5% received an opioid prescription within 90 days after discharge. Substantial differences in opioid prescribing and dosing have been observed across various patient populations based on race and geographic locations (14–16). However, nation wide claims-based studies on variations in opioid prescribing and utilization following orthopaedic trauma have not been published to date. Previous estimates of opioid prescribing after orthopaedic trauma vary widely, between 4.3–68.4% (13, 21). Uniquely, this work builds upon previous research that has been limited to single institutional investigations or retrospective reviews based on a single anatomical injury site or orthopaedic surgery type (12, 13, 21, 27). In this national sample, substantial differences in opioid receipt and dosage were observed based on patient demographics and clinical characteristics.

Receipt of an opioid prescription after injury differed across patient populations in this sample. Most notably, inequities in opioid receipt were noted among Black and Hispanic patients compared to white patients even when adjusting for acuity (e.g., length of stay). Black patients in this sample were observed to have 13% lower odds ratio compared to white patients in receiving an opioid prescription after injury while Hispanic patients had a 28% lower odds ratio. Further, Black and Hispanic patients received lower doses of medication compared to injured white Medicaid beneficiaries. These findings reflect those by other investigators utilizing Medicare claims (16) where Black and white patients were found to have similar receipt of opioid prescriptions, yet Black patients received 36% lower doses. Findings from the present analysis underscore that stigma may persist when dispensing opioids to patients in need of analgesia (28) after injury. Racial bias by clinicians in the assessment and management of pain, specifically towards Black patients, is well documented in other clinical settings and hinders equitable access to opioid prescriptions (29, 30). Findings illustrate, for the first time, possible inequitable prescribing exists, nationally, among clinicians caring for patients on Medicaid after orthopaedic injury. However, future research examining prescribing in samples with a variety of insurance coverage (e.g., Medicaid, private claims, etc.) are warranted to better discern prescribing patterns after injury while accounting for biological, environmental, and social factors that may influence prescribing.

Other notable differences in opioid prescribing were seen based on patients’ sex, age, length of hospitalization, as well as year and location at time of injury. In outpatient settings both female patients and those older than 25 have been found to have a greater likelihood of filling their opioid prescriptions (31). To date, this trend has not been examined following orthopaedic trauma. In the present study, injured male Medicaid beneficiaries had 12% higher odds ratio of receiving an opioid compared to females. Compared to those aged <31, adults aged 31–50 had a 15% higher odds ratio of being prescribed an opioid after injury, but at lower doses. Surgical team prescribing has been found to vary by length of stay, with longer hospitalization linked to higher doses (32). While no difference in doses were observed based on length of stay in this analysis, patients with longer hospitalizations had decreased odds ratio of receiving an opioid prescription. In other non-trauma specific studies, patients with disabilities were found to receive higher incidence of continuous opioid use and significantly greater amounts (33). These differences by patient characteristics indicate the unique differences in opioid prescribing, and potentially pain management needs, after injury that may not be reflective of larger non-diagnosis specific analyses. Evidence-based opioid prescribing guidelines have been found to reduce the quantity of opioids prescribed after surgery but less is known on whether they reduce inequities in prescribing while optimizing pain outcomes (34, 35). National guidelines for use of opioids in adults after trauma hospitalization that also highlight the utilization of medications and nonpharmacological interventions, may help limit practice variation, and reduce implicit bias and potential harms in opioid usage.

Prescribing and dosing of opioid medication for injured patients in this national dataset changed over time. Despite the decline in the odds ratio of receiving an opioid prescription over time, the average MME increased from 2015–2018. These findings may reflect restrictions in prescribing seen across specialties, nationally, following the release of the 2016 CDC Guidelines for Prescribing Opioids for Chronic Pain (36). While receipt of opioids did not differ by region in this sample, dosing was significantly lower in the Southeast and higher in the Northeast. Other investigators have found national declines in opioid prescribing and dosage across clinical settings but note substantial variation in state level prescribing patterns persist (31, 37). To date, regional differences in prescribing across trauma patient populations have not been examined. These findings show the potential utility of tailored trauma specific pain management guidelines to facilitate equitable prescribing by surgical teams to injured patients during and after hospitalization.

There are limitations to this analysis. Because this study was conducted using an administrative claims database, we did not have a control group. We are unable to observe a range of other factors which may be associated with the outcomes of interest; these include measures of clinical injury severity and the state of each adult’s environment that could directly or indirectly impact opioid utilization. Causality cannot be inferred from this analysis. Another limitation is the study period, not including the most recent years; we have no *a priori* reason, however, to believe that the associations of interest would have changed since the timeframe we examined in the data (38). During the study period, the Medicaid claims data have experienced changes in the data format from MAX, MAX-T to TAF files (39, 40), changes in the diagnosis coding (ICD-9 and ICD-10) (41) and changes in the procedure codes (42). Our diagnosis and procedure coding captured much of the study population, but may have missed some trauma cases, potentially adding bias in our sample because several states or regions may have been faster in accurately coding diagnoses and procedures. Further, this study was unable to control for potential comorbidities that may influence prescribers. Because of the changes in recording the Medicaid claims data, some of the factors in our analysis include null values for zip code and race/ethnicity. We have created algorithms to capture this information, but several states have large percentages of missing values particularly for more recent years (43). Medicaid claims data accounted for fulfilled prescriptions and relied on the conversion of prescriptions to MME for analysis, hence it is not possible to determine the appropriateness of opioid prescriptions nor the actual use of opioids in the study. The Medicaid dataset did not include acuity, such as Injury Severity Score, but the analyses included hospitalization stay as a proxy for severity. Future research incorporating severity metrics is warranted. While findings are generalizable to injured Medicaid beneficiaries, future research with commercial claims is needed to determine if trends are consistently observed across traumatically injured patient populations regardless of insurance status. Despite these limitations, this study is among the first to leverage national data over a longitudinal period to elucidate prescribing trends across injured populations with Medicaid coverage.

## 5 Conclusion

This study is the first to leverage a national data repository to examine longitudinal opioid prescribing trends across patient populations and regions following orthopaedic trauma. Injured patients with disabilities, prolonged hospitalizations, and both Black and Hispanic patients, relative to white patients, were less likely to receive an opioid prescription. Further, Black and Hispanic patients received lower opioid doses compared to white patients after injury. These inequitable differences in opioid prescribing persists nationally after orthopaedic injury among adult Medicaid beneficiaries. Research is needed to elucidate nuances in prescribing differences and inform the development of scalable interventions, such as guidelines, to mitigate inequities in opioid prescribing practices after injury.

## Data availability statement

The datasets presented in this article are not readily available because this project used deidentified data from the Centers for Medicare and Medicaid Services Health Services. Data from the Centers for Medicare and Medicaid Services does not belong to the authors but is provided to the authors through a Data Use Agreement. Data sharing will be at the discretion of Centers for Medicare and Medicaid Services and will require a specific data use agreement between third party interested data users and Centers for Medicare and Medicaid Services. The authors are committed to work with anybody interested in accessing the data to facilitate the process. Requests to access the datasets should be directed to NS, nicoleta.serban@isye.gatech.edu.

## Ethics statement

The studies involving humans were approved by Georgia Institute of Technology IRB. The studies were conducted in accordance with the local legislation and institutional requirements. Written informed consent for participation was not required from the participants or the participants’ legal guardians/next of kin in accordance with the national legislation and institutional requirements.

## Author contributions

NG: Investigation, Writing – original draft, Writing – review & editing. GZ: Data curation, Formal analysis, Investigation, Methodology, Software, Writing – review & editing. MK: Data curation, Formal analysis, Investigation, Methodology, Software, Writing – review & editing. MS: Funding acquisition, Investigation, Writing – review & editing. YW: Writing – review & editing. CR: Funding acquisition, Writing – review & editing. NS: Conceptualization, Data curation, Formal analysis, Funding acquisition, Investigation, Methodology, Project administration, Resources, Software, Supervision, Writing – original draft, Writing – review & editing.
