# Supplementary material for: Opioid utilization after orthopaedic trauma hospitalization among Medicaid-insured adults
Source: Front Public Health. 2024 Mar 26;12:1327934. doi: 10.3389/fpubh.2024.1327934 (PMC11003548; doi:10.3389/fpubh.2024.1327934)
Supplement: Supplementary file 1 [file Table_1.docx]

Online Supplemental Material

# Online-Supplement A: Study Population Identification & Feature Extraction

The study population was drawn from MAX and TAF IP claims data of patients between aged 18 to 65 from 2010-2018. The specific procedure followed for data extraction is summarized below:

1. All claims matching the Trauma CPT codes lists (refer to [Table S6](#tables6_cpt)) from Emory Medical Care Foundation - Orthopaedic Surgery were selected from the MAX and TAF IP files.
2. Prescription records for eligible trauma-diagnosed patients were extracted from the Medicaid RX Prescription Drug file using patient-personal information such as patient identification number, billing state, prescription filling date, prescription days, National Drug Code (NDC), and Type of service.
3. Information regarding MME (Morphine Milligram Equivalent) was extracted from the CDC MME Translation Table based on the National Drug Code (NDC) in the prescription.
   1. CDC MME Translation Table includes specific drug name, Unit of Measure, Class (opioid or non-opioid), Strength per Unit, and MME conversion factor.
4. The daily MME was calculated by combining the prescription information with the MME conversion factor.
   1. The formula used was: Strength per Unit X (Number of Units / Days Supply) X MME conversion factor = MME/Day
   2. ***Note*:** For fentanyl patches, since they remain in place for 3 days, the Days Supply is calculated by multiplying the number of patches by three.
5. Demographic information of eligible patients was obtained from the MAX/TAF file, including Date of Birth, Date of Death, State code, Gender, Race, County code, Eligibility group codes for all 12 months of the reference year, Service Start Date, and Service End Date.
   1. Patients with a death indicator were excluded based on Date of Death.
   2. The age calculation was based on the start date of the latest hospitalization.
   3. The State code encompasses the fifty states of the United States, along with Puerto Rico and the Virgin Islands.
   4. The FIPS code was generated based on the patient's billing state code and county code. The Rural-Urban Continuum Codes were used to categorize the patient's metropolitanization
      1. metro region (RUCC index < =3), nonmetro region (RUCC index >=4), NA (if RUCC index was not found).
   5. Eligibility group codes (refer to Table [S7](#tables7_eli_taf) and [S8](#tables7_eli_taf)) for all 12 months were used to classify patients. Pregnant women and children were removed, followed by categorizing patients as Disabled if codes related to disabilities were found. Otherwise, patients were classified as Income based.
6. The unique patient prescription information (steps 1 to 3) was merged with the demographic information (step 4).
   1. The most recent hospitalization demographic information was considered the baseline for patients with multiple hospitalizations.

Supplemental Table 1: CPT Codes Used to Identify Trauma Services

| Codes |  |
| --- | --- |
| **Amputation** | 24900, 24920, 25907, 25909, 27590, 27592, 27594, 27596, 27598, 27880, 27882, 27884, 27886, 27888 |
| **External fixation** | 20690, 20692, 20693, 20696, 20697 |
| **Nonunion/Malunion** | 23485, 24400, 24430, 25400, 25405, 25415, 25420, 25425, 25426, 27161, 27165, 27170, 27450, 27470, 27472, 27705, 27707, 27709, 27720, 27724, 27725 |
| **Shoulder and Humerus** | 23472, 23515, 23530, 23532, 23550, 23552, 23585, 23615, 23616, 23630, 23660, 23670, 23680, 23920, 24515, 24516, 24538, 24545,  24546, 24566, 24575, 24579, 23582, 24586,  24587, 24615, 24635, 24665, 24666, 24685 |
| **Forearm and Wrist** | 25310, 25400, 25405, 25515, 25525, 25526,  25545, 25574, 25575, 25606, 25607, 25608, 25609, 25628, 25645, 25652,25670, 25671,  25676, 25685, 25695 |
| **Pelvis and acetabulum** | 27215, 27216, 27217, 27218, 27226, 27227,  27228 |
| **Femur** | 27236, 27244, 27245, 27248, 27253, 27254, 27386, 27506, 27507, 27511, 27513, 27514, 27519, 27524 |
| **Knee and Tibia** | 27535, 27536, 27540, 27556, 27557, 27558, 27756, 27758, 27759, 27766, 27769, 27784, 27792, 27814, 27822, 27823, 27826, 27827, 27828 |
| **Foot and ankle** | 27244, 27245, 27248, 27253, 27254, 27386, 27506, 27507, 27511, 27513, 27514 |

Supplemental Table 2: Grouping of Medicaid Eligibility Codes (TAF)

| **Eligibility Codes** | **Grouping** |
| --- | --- |
| 02, 12, 15, 16, 19, 20, 21, 24, 34, 36, 37, 39, 42, 43, 44, 46, 51, 52, 56, 59, 60, 69 | Disabled |
| 05, 06, 07, 08, 09, 22, 29, 30, 31, 45, 50, 53, 54, 55, 61, 62, 63, 64, 65, 66, 68, 70 | Children or Pregnant Woman |
| All other codes | Income Based |

Supplemental Table 3: Grouping of Medicaid Eligibility Codes (MAX)

| **Eligibility Codes** | **Grouping** |
| --- | --- |
| 12, 22, 32, 3A, 42, 52, | Disabled |
| 14, 16, 24, 34, 48, 44, 54 | Children |
| All other codes | Income Based |

Supplemental Table 4: Characteristics of Opioid Utilization After Trauma Hospitalization in the Medicaid-insured Adults

|  | Eligible trauma related patients  [n=86,091] | Filled opioids within 1 month of discharge  [n=30,698] | Filled opioids within 3 months of discharge  [n=19,654] |
| --- | --- | --- | --- |
|  |  |  |  |
| *Demographics* |  |  |  |
| Age (18-30) (reference group), *%* | 14241, 16.54% | 5236, 17.06% | 2553, 12.99% |
| Age (31-50), *%* | 32683, 37.96% | 12699, 41.37% | 7962, 40.51% |
| Age (51-65), *%* | 39167, 45.50% | 12763, 41.57% | 9139, 46.5% |
| Female (reference group), % | 44077, 51.2% | 15033, 48.97% | 10063, 51.2% |
| Male, % | 42014, 48.8% | 15665, 51.03% | 9591, 48.8% |
| Race/ethnicity |  |  |  |
| White (reference group), % | 42946, 49.88% | 15806, 51.49% | 10651, 54.19% |
| Black, % | 17076, 19.83% | 5722, 18.64% | 3698, 18.82% |
| Hispanic, % | 9655, 11.21% | 3024, 9.85% | 1671, 8.5% |
| Other, % | 3547, 4.12% | 1251, 4.08% | 666, 3.39% |
| Unknown race/ethnicity, % | 12867, 14.96% | 4895, 15.94% | 2968, 15.1% |
| Rurality-Urbanicity |  |  |  |
| Metro counties (reference group), % | 66402, 77.13% | 23207, 75.6% | 14743, 75.02% |
| Nonmetro counties, % | 14466, 16.8% | 5282, 17.2% | 3556, 18.09% |
| Unknown, % | 5223, 6.07% | 2209, 7.2% | 1355, 6.89% |
| Region |  |  |  |
| Northeast, % | 19522, 22,68% | 6981, 22.74% | 4139, 21.06% |
| Southeast, % | 16706, 19.41% | 5588, 18.2% | 4028, 20.49% |
| Midwest (reference group), % | 21646, 25.14% | 8017, 26.12% | 5320, 27.07% |
| West, % | 18844, 21.89% | 6904, 22.49% | 3928, 19.99% |
| Southwest, % | 9373, 10.88% | 3208, 10.45% | 2239,11.39% |
| Year of Hospitalization, % |  |  |  |
| 2010-2012 (reference group) , % | 23368, 27.14% | 5350, 17.43% | 4444, 22.61% |
| 2013-2014, % | 10188, 11.83% | 3258, 10.61% | 2661, 13.54% |
| 2015-2016, % | 21840, 25.37% | 9022, 29.39% | 5759, 29.3% |
| 2017-2018, % | 30695, 35.66% | 13068, 42.57% | 6790, 34.55% |
| *Length of stay,* mean | 7.03 | 6.65 | 7.03 |
| *Multiple services*, % | 4563, 5.3% | 1671, 5.44% | 1230, 6.26% |
| *Eligibility* |  |  |  |
| Income, % | 52507, 60.99% | 21731, 70.79% | 12390, 63.04% |
| Disability, % | 33584, 39.01% | 8967, 29.21% | 7264, 36.96% |

Notes: Medicaid patients who had received at least one opioid prescription represented 43% of the total study population.

Supplemental Table 5: Statistical Significance Comparison: Binary Outcome vs Opioid Days-Supply vs Opioid Duration

|  | | Medicaid-enrolled patients with opioid prescription(s) [n=60,462] | | | | | | |
| --- | --- | --- | --- | --- | --- | --- | --- | --- |
|  | | Opioid Use-Binary Outcome Analysis  [30 days] [90 days] | | Opioid Days-Supply Outcome Analysis  [30 days] [90 days] | | Opioid Dosage Outcome Analysis  [30 days] [90 days] | | |
|  | |  |  |  |  |  | |  |
| Age |  | |  |  |  |  | |  |
| Age (31-50) | [+][97] | | [+][100] | [+][100] | [+][100] | [-][55] | [-][97] | |
| Age (51-65) | [-][4] | | [+][100] | [+][100] | [+][100] | [-][100] | [-][100] | |
| Male | [+][100] | | [+][3] | [+][2] | [-][3] | [+][41] | [+][99] | |
| Race/ethnicity |  | |  |  |  |  |  | |
| Black | [-][99]] | | [-][99] | [-][100] | [-][100] | [-][93] | [-][97] | |
| Hispanic | [-][100] | | [-][100] | [-][100] | [-][100] | [-][100] | [-][100] | |
| Other | [-][57] | | [-][100] | [-][99] | [-][90] | [+][18] | [+][8] | |
| Unknown | [-][93] | | [-][92] | [-][83] | [-][71] | [-][28] | [-][53] | |
| Rurality-Urbanicity |  | |  |  |  |  |  | |
| Nonmetro counties | [+][81] | | [+][24] | [+][3] | [+][21] | [-][5] | [-][14] | |
| Unknown | [+][46] | | [+][88] | [+][96] | [+][94] | [-][100] | [-][99] | |
| Region |  | |  |  |  |  |  | |
| Southeast | [-][25] | | [+][5] | [-][9] | [-][11] | [-][93] | [-][91] | |
| Northeast | [-][12] | | [-][99] | [-][37] | [-][43] | [+][100] | [+][100] | |
| West | [+][3] | | [-][96] | [-][4] | [-][9] | [+][8] | [+][41] | |
| Southwest | [+][2] | | [+][4] | [+][1] | [+][1] | [-][19] | [-][8] | |
| Year of Hospitalization |  | |  |  |  |  |  | |
| 2013-2014 | [+][100] | | [+][100] | [-][47] | [-][2] | [+][26] | [+][6] | |
| 2015-2016 | [+][100] | | [+][100] | [-][53] | [-][23] | [+][99] | [+][98] | |
| 2017-2018 | [+][100] | | [+][96] | [-][100] | [-][100] | [+][100] | [+][100] | |
| Length of stay | [+][97] | | [+][2] | [+][100] | [+][100] | [-][18] | [-][4] | |
| History of traumatic hospitalization | [+][34] | | [+][100] | [+][100] | [+][98] | [+][2] | [-][2] | |
| Eligibility |  | |  |  |  |  |  | |
| Disability | [-][100] | | [-][80] | [+][84] | [+][96] | [-][33] | [-][5] | |

Notes: +: positive estimated coefficient, -: negative estimated coefficient, % of p-values <0.05 [##]

Supplemental Table 6: Opioid Use Binary Outcome Analysis with after discharge three months opioid-monitoring period ^a^ (N= 86,091)

|  | Odds  Ratio | 95% CI lower Bound | 95% CI Upper Bound | Percent of p-values <0.05 |
| --- | --- | --- | --- | --- |
| *Demographics* |  |  |  |  |
| Age (31-50) | 1.467 | 1.367 | 1.583 | 100 |
| Age (51-65) | 1.405 | 1.311 | 1.519 | 100 |
| Male | 1.003 | 0.953 | 1.051 | 3 |
| Race/ethnicity |  |  |  |  |
| Black | 0.848 | 0.79 | 0.914 | 99 |
| Hispanic | 0.667 | 0.603 | 0.728 | 100 |
| Other | 0.704 | 0.618 | 0.812 | 100 |
| Unknown race/ethnicity | 0.87 | 0.805 | 0.935 | 92 |
| Rurality-Urbanicity |  |  |  |  |
| Nonmetro counties | 1.054 | 0.989 | 1.129 | 24 |
| Unknown | 1.19 | 1.082 | 1.317 | 88 |
| Region | - | - | - | - |
| Southeast | 1.026 | 1.04 | 1.101 | 5 |
| Northeast | 0.842 | 0.782 | 1.11 | 99 |
| West | 0.864 | 0.8 | 0.93 | 96 |
| Southwest | 1.028 | 0.954 | 1.115 | 4 |
| Year of Hospitalization |  |  |  |  |
| 2013-2014 | 1.545 | 1.41 | 1.704 | 100 |
| 2015-2016 | 1.48 | 1.357 | 1.605 | 100 |
| 2017-2018 | 1.191 | 1.088 | 1.289 | 96 |
| *Length of stay* | 0.994 | 0.99 | 0.997 | 2 |
| *Eligibility criteria* |  |  |  |  |
| Disability | 0.9 | 0.838 | 0.953 | 80 |
| *History of traumatic hospitalization* | 1.269 | 1.14 | 1.415 | 100 |

*Notes*: CI=confidence interval.

^a^A Logistic regression was used to examine how the explanatory factors explain the odds ratio of opioid prescription filled versus not filled within the opioid-monitoring period. To correct for ‘inflated statistical significance’ due to a large sample size, the regression was estimated in 100 sub-samples that included 30% of the population. The mean odds ratio is presented from these 100 replicates, and the 95% confidence interval is derived using the 2.5^th^ percentile for the lower bound and the 97.5th percentile for the upper bound of the estimated odds ratio across the 100 model replicates. The percent of p-values for each covariate that were significant in these 100 sub-samples is presented in the far-right column.

Supplemental Table 7: Opioid Duration Outcome Analysis^a^ with recorded opioid use within the three-months opioid-monitoring period. (N=36,553)

|  | Rate Ratio | 95% CI lower Bound | 95% CI Upper Bound | Percent of p-values <0.05 |
| --- | --- | --- | --- | --- |
| *Demographics* |  |  |  |  |
| Age (31-50) | 1.187 | 1.13 | 1.244 | 100 |
| Age (51-65) | 1.223 | 1.163 | 1.285 | 100 |
| Male | 0.992 | 0.966 | 1.017 | 3 |
| Race/ethnicity |  |  |  |  |
| Black | 0.895 | 0.864 | 0.93 | 100 |
| Hispanic | 0.868 | 0.827 | 0.909 | 100 |
| Other | 0.87 | 0.799 | 0.947 | 90 |
| Unknown race/ethnicity | 0.935 | 0.893 | 0.983 | 71 |
| Rurality-Urbanicity |  |  |  |  |
| Nonmetro counties | 1.025 | 0.989 | 1.071 | 21 |
| Unknown | 1.109 | 1.051 | 1.17 | 94 |
| Region |  |  |  |  |
| Southeast | 0.982 | 0.936 | 1.022 | 11 |
| Northeast | 0.959 | 0.916 | 1.004 | 43 |
| West | 0.981 | 0.942 | 1.017 | 9 |
| Southwest | 1.002 | 0.947 | 1.052 | 1 |
| Year of Hospitalization |  |  |  |  |
| 2013-2014 | 0.991 | 0.944 | 1.045 | 2 |
| 2015-2016 | 0.961 | 0.914 | 1.017 | 23 |
| 2017-2018 | 0.788 | 0.749 | 0.834 | 100 |
| *Length of stay* | 1.006 | 1.004 | 1.008 | 100 |
| *Eligibility criteria* |  |  |  |  |
| Disability | 1.089 | 1.045 | 1.138 | 98 |
| *History of traumatic hospitalization* | 1.129 | 1.062 | 1.206 | 96 |

*Notes*: CI=confidence interval.

^a^ Negative Binomial regression was used to examine how the explanatory factors describe the variability in the count of days-supply for patients with recorded opioid use within the opioid-monitoring period. To correct for ‘inflated statistical significance’ due to a large sample size, the regression was estimated in 100 sub-samples that included 30% of the population. The Rate Ratio presented from these 100 replicates, and the 95% confidence interval is derived using the 2.5^th^ percentile for the lower bound and the 97.5th percentile for the upper bound of the predicted difference in visits across the 100 model replicates. The percent of p-values for each covariate that were significant in these 100 sub-samples is presented in the far-right column.

Supplemental Table 8**:** Opioid Dosage Outcome^a^ with recorded opioid use within the three-months opioid-monitoring period (N=36,553)

|  | Coefficients | 95% CI lower Bound | 95% CI Upper Bound | Percent of p-values <0.05 |
| --- | --- | --- | --- | --- |
| *Demographics* |  |  |  |  |
| Age (31-50) | -0.103 | -0.147 | -0.063 | 97 |
| Age (51-65) | -0.22 | -0.271 | -0.174 | 100 |
| Male | 0.074 | 0.044 | 0.107 | 99 |
| Race/ethnicity |  |  |  |  |
| Black | -0.108 | -0.163 | -0.058 | 97 |
| Hispanic | -0.178 | -0.244 | -0.116 | 100 |
| Other | 0.052 | -0.04 | 0.135 | 8 |
| Unknown race/ethnicity | -0.06 | -0.11 | -0.011 | 53 |
| Rurality-Urbanicity |  |  |  |  |
| Nonmetro counties | -0.029 | 0.084 | 0.017 | 14 |
| Unknown | -0.182 | -0.244 | -0.115 | 99 |
| Region |  |  |  |  |
| Southeast | -0.095 | -0.14 | -0.047 | 91 |
| Northeast | 0.166 | 0.116 | 0.21 | 100 |
| West | 0.053 | 0.007 | 0.105 | 41 |
| Southwest | -0.326 | -0.094 | 0.03 | 8 |
| Year of Hospitalization |  |  |  |  |
| 2013-2014 | 0.025 | -0.049 | 0.084 | 6 |
| 2015-2016 | 0.153 | 0.083 | 0.205 | 98 |
| 2017-2018 | 0.271 | 0.209 | 0.326 | 100 |
| *Length of stay* | -0.000 | 0.003 | 0.003 | 4 |
| *Eligibility criteria* |  |  |  |  |
| Disability | -0.017 | -0.066 | 0.033 | 5 |
| *History of traumatic hospitalization* | -0.001 | -0.08 | 0.07 | 2 |

*Notes*: CI=confidence interval.

^a^ Linear regression was used to examine how the explanatory factors explain the variability in the log of the average MME per-day for patients with recorded opioid use within the opioid-monitoring period. To correct for ‘inflated statistical significance’ due to a large sample size, the regression was estimated in 100 sub-samples that included 30% of the population.

The Coefficients is presented from these 100 replicates, and the 95% confidence interval is derived using the 2.5^th^ percentile for the lower bound and the 97.5th percentile for the upper bound of the predicted difference in visits across the 100 model replicates. The percent of p-values for each covariate that were significant in these 100 sub-samples is presented in the far right column.
